# Optimal Treatment Strategy in Rectal Cancer Surgery: Should We Be Cowboys or Chickens?

**DOI:** 10.1245/s10434-015-4385-7

**Published:** 2015-02-18

**Authors:** Heleen S. Snijders, Nicoline J. van Leersum, Daan Henneman, Alexander C. de Vries, Rob A. E. M. Tollenaar, Anne M. Stiggelbout, Michel W. J. M. Wouters, Jan Willem T. Dekker

**Affiliations:** 1 Department of Surgery, Leiden University Medical Centre, Leiden, The Netherlands; 2 Department of Surgery, Medical Center Haaglanden, The Hague, The Netherlands; 3 Department of Medical Decision Making, Leiden University Medical Center, Leiden, The Netherlands; 4 Department of Surgery, Netherlands Cancer Institute-Antoni van Leeuwenhoek Hospital, Amsterdam, The Netherlands; 5 Department of Surgery, Reinier de Graaf Gasthuis, Delft, The Netherlands

## Abstract

**Background and Purpose:**

Surgeons and hospitals are increasingly accountable for their postoperative complication rates, which may lead to risk adverse treatment strategies in rectal cancer surgery. It is not known whether a risk adverse strategy leads to providing better care. In this study, the association between the strategy of hospitals regarding defunctioning stoma construction and postoperative outcomes in rectal cancer treatment was evaluated.

**Methods:**

Population-based data of the Dutch Surgical Colorectal Audit, including 3,104 patients undergoing rectal cancer resection between January 2009 and July 2012 in 92 hospitals, were used. Hospital variation in (case-mix-adjusted) defunctioning stoma rates was calculated. Anastomotic leakage and 30-day mortality rates were compared in hospitals with a high and low tendency towards stoma construction.

**Results:**

Of all patients, 76 % received a defunctioning stoma; 9.6 % of all patients developed anastomotic leakage. Overall postoperative mortality rate was 1.8 %. The hospitals’ adjusted proportion of defunctioning stomas varied from 0 to 100 %, and there was no significant correlation between the hospitals’ adjusted stoma and anastomotic leakage rate. Severe anastomotic leakage was similar (7.0 vs. 7.1 %; *p* = 0.95) in hospitals with the lowest and highest stoma rates. Mild leakage and postoperative mortality rates were higher in hospitals with high stoma rates.

**Conclusions:**

A high tendency towards stoma construction in rectal cancer surgery did not result in lower overall anastomotic leakage or mortality rates. It seems that the ability to select patients for stoma construction is the key towards preferable outcomes, not a risk adverse strategy.

Surgical resection is the cornerstone of rectal cancer treatment. If tumor size, stage, and location allow for a sphincter-preserving resection, and bowel continuity is restored, the surgeon has to decide whether or not to defunction the anastomosis. The advantage of a defunctioning stoma can be that it decreases the consequences of anastomotic leakage, and may also decrease its incidence.^1^,^2^ Anastomotic leakage is a serious complication causing reoperation, prolonged hospital stay, morbidity, mortality, and possibly worse oncological outcome^3^–^5^ On the other hand, a stoma has evident disadvantages; defunctioning stomas can induce morbidity, discomfort (decreased quality of life), higher costs,^6^ longer hospitalization,^7^ and even mortality from surgery to close the stoma^8^–^12^ Furthermore, 80 % of defunctioning stomas are only reversed after 4 months, and 20 % are never reversed.^13^

Nowadays, quality of care has become a major topic, and surgeons and hospitals are increasingly accountable for their postoperative complication rates. This may lead to risk adverse treatment strategies. Previous research suggests that differences in professional opinion may lead to variation in healthcare delivery^14^–^21^ The threshold for the decision to construct a stoma to avoid the risk for anastomotic leakage may also vary between surgeons. Some surgeons may be bigger risk takers or more risk adverse than others. However, attempts to avoid or limit the risk for anastomotic leakage after colorectal surgery by frequent use of stomas is only in the patient’s interest if it in fact lowers clinically relevant anastomotic leakage and mortality rates.

The objective of this study was to investigate whether hospitals differ in their treatment strategy regarding construction of defunctioning stomas in rectal cancer surgery, and to assess if a hospital’s treatment strategy is related to its postoperative outcomes, such as clinically relevant anastomotic leakage and mortality rates.

## Methods

### Study Cohort

Data were derived from the Dutch Surgical Colorectal Audit (DSCA). The DSCA contains data registered by 92 hospitals (representing all hospitals performing colorectal cancer surgery in The Netherlands), and over 90 % of all eligible patients are included. The dataset is disease-specific for colorectal cancer and has shown a nearly 100 % concordance on most items upon validation against the Netherlands Cancer Registry dataset.^22^ All patients having undergone anterior resection for primary rectal cancer between 1 January 2009 and 31 July 2012 were evaluated. Minimal data requirements for inclusion in the analysis were information on tumor location, date of surgery, and mortality. Patients without an anastomosis, with metastasis at the time of primary surgery, resections for multiple synchronous colorectal tumors, and with a tumor less than 5 cm from the anal verge were excluded because these represent subgroups of patients with specific treatment perspectives and subsequent different expected outcomes.

### Definitions

Overall anastomotic leakage, as used in the hospital comparisons, was defined as ‘clinically relevant anastomotic leak requiring a re-intervention, either radiological (mild) or surgical (severe)’. Postoperative mortality was defined as ‘in-hospital mortality or all deaths within 30 days after primary surgery’.

The following case-mix factors were considered: age, sex, American Society of Anesthesiologists (ASA) classification, abdominal surgical history, tumor height, preoperative tumor complications, and urgency of the resection. Considered treatment factors were surgical procedure (laparoscopic or open), and neoadjuvant treatment.

Hospitals were stratified into non-teaching and teaching hospitals. Procedural volume in rectal cancer resections was calculated for each hospital before the aforementioned exclusion of patients, and categorized into <25, 25–50 and >50 resections per year.

## Statistical Considerations

As patient- and tumor-related case-mix factors may be responsible for a large part of the hospital variation in the proportion of patients with a defunctioning stoma, we adjusted for these differences by calculating the observed/expected (O/E) stoma rate. The observed outcome was the number of patients with a defunctioning stoma in a hospital, and the expected outcome was the sum of all patients’ estimated probabilities for a defunctioning stoma. Patients’ probability estimates were derived from a backwards stepwise multivariate logistic regression model, fitted on the data of all included hospitals, and using all case-mix factors mentioned above. For an average performing hospital, the observed outcome will be equal to the expected outcome, resulting in an O/E outcome ratio of 1. Hospitals that construct more defunctioning stomas than average have an O/E outcome ratio higher than 1, while this ratio is lower than 1 in hospitals with lower than average stoma rates.

The adjusted O/E ratios of the hospitals were plotted against their anastomotic leakage rates.

The relation between the hospitals’ strategy and its outcomes was analyzed by two methods. First, to evaluate whether stoma rates were related to (lower) anastomotic leakage rates on a hospital level, a linear correlation was calculated using Pearson’s correlation coefficient *R.* Second, to evaluate whether a risk adverse strategy (high stoma rates) is related to better postoperative outcomes on a hospital level, hospitals were grouped into equally-sized groups based on quintiles of their case-mix-adjusted rate of defunctioning stomas.

Differences between groups in outcomes (mild and severe anastomotic leakage and mortality rates) were analyzed using a Chi square test.

The association of patient- and tumor-related case-mix factors, hospital factors (teaching status, volume), and treatment factors (neoadjuvant therapy, laparoscopic surgery) with being in the high stoma group was assessed with a Chi square test and multivariate logistic regression analysis, considering the same case-mix factors as mentioned above. All statistical analyses were performed in PASW Statistics, IBM Corporation (previously SPSS Software, Armonk, NY, USA).

## Results

Between 1 January 2009 and 31 July 2012, a total of 92 hospitals registered all rectal cancer patients in the DSCA. After exclusion of ineligible patients, a total of 3,104 patients were included in the analysis. Characteristics of the included patients and hospitals are shown in Table 1.

**Table 1:** Patient, tumor, and treatment characteristics of included patients

	*N* (%)
Age [mean (range)]	66 (15–97)
Male sex	1,850 (60)
ASA classification	
I–II	2,567 (83)
III+	369 (12)
Missing	168 (5)
Abdominal surgical history, yes	808 (26)
Tumor location (cm)	
≥10	1,149 (14)
<10	1,660 (20)
Urgency, acute/urgent	57 (2)
Tumor stage	
(Y) pT0/X	207 (7)
pT1	269 (9)
pT2	990 (32)
pT3	1,533 (49)
pT4	105 (3)
Surgical preoperative treatment	
Stoma	162 (5)
Stent	8 (0.3)
Other	51 (3)
Neoadjuvant treatment	
5 × 5 Gy	1,623 (52)
Chemoradiation	825 (27)
Surgical procedure	
Laparoscopic resection	1,393 (45)
Hospital type	
Teaching hospital	2,175 (70)
Non-teaching hospital	929 (30)
Hospital volume	
High (>50/year)	875 (28)
Medium (25–50/year)	1,490 (48)
Low (<25/year)	739 (24)

*ASA* American Society of Anesthesiologists risk score

Overall, 67 % (*n* = 2,080) of all patients received an anastomosis with a defunctioning stoma.

In total, 302 patients (9.6 %) developed anastomotic leakage. The majority (187 of 302, 62 %) were severe leakages requiring surgical reintervention. Anastomotic leakage rates were somewhat higher in patients with a defunctioning stoma (9.3 vs. 10.4 %), but this difference was not statistically significant (*p* = 0.35). Fifteen of 302 patients who developed anastomotic leakage died during their hospital stay or within 30 days after surgery (5 %). Overall postoperative mortality rate was 1.8 % (*n* = 187); anastomotic leakage caused one-quarter of overall mortality. There was no difference in overall mortality rate between both groups—1.3 % in patients without stoma versus 2.1 % in patients with stoma (*p* = 0.11).

### Hospitals

Relevant case-mix factors were selected by backward stepwise logistic regression analysis. Relevant factors for the proportion of defunctioning stomas were sex, preoperative complications, tumor location, and laparoscopic surgery.

**Fig. 1:**
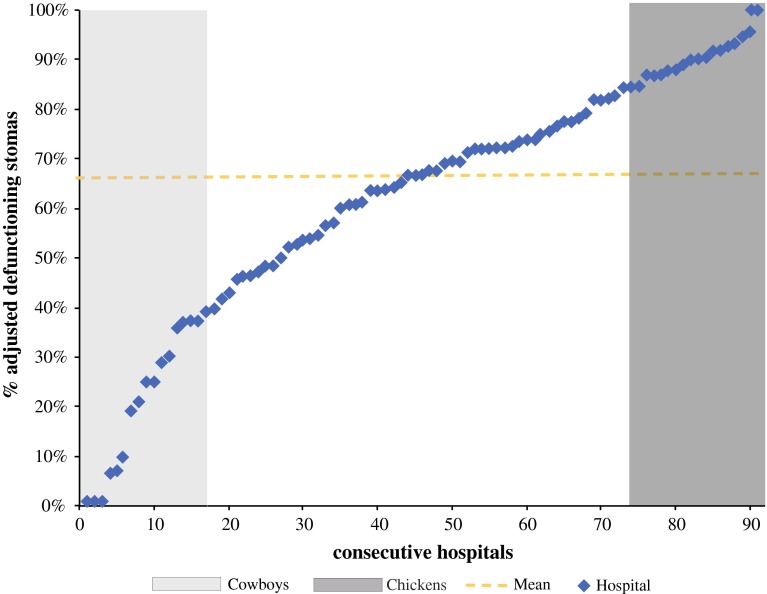
Hospitals ranked by their case-mix-adjusted defunctioning stoma rate. Based on quintiles, groups of low (*left*) and high (*right*) stoma rates were identified

**Fig. 2:**
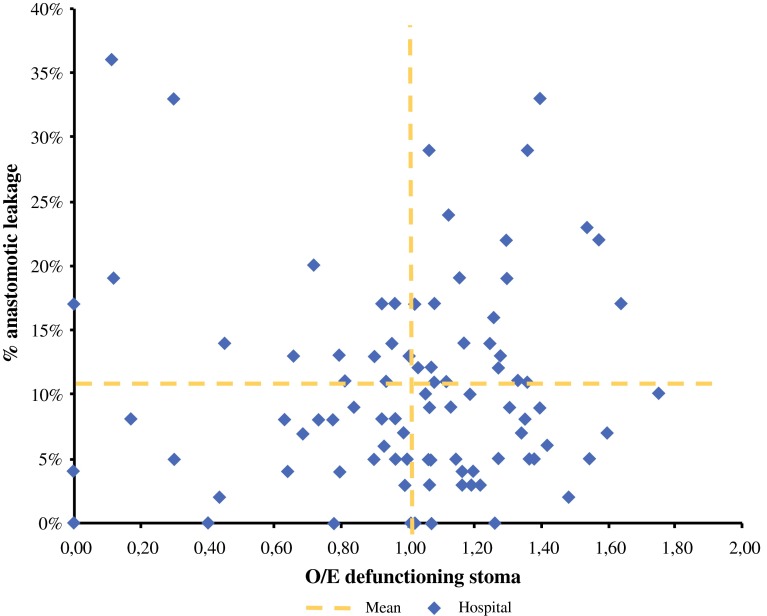
Adjusted defunctioning stoma O/E rates of hospitals, plotted against their anastomotic leakage rates. *O/E* observed/expected

The hospitals’ unadjusted proportion of defunctioning stomas varied considerably: percentages ranged from 0 to 100 % (Fig. 1). Figure 2 shows the relation between the hospitals’ adjusted proportion (O/E ratio) of defunctioning stomas and their overall anastomotic leakage rate (which varied from 3 to 18 %). There was a weak positive correlation between the hospitals’ adjusted O/E stoma ratio and anastomotic leakage rates (*r* = 0.032), although this was not statistically significant (*p* = 0.76).

### Low Versus High Stoma Rate

**Fig. 3:**
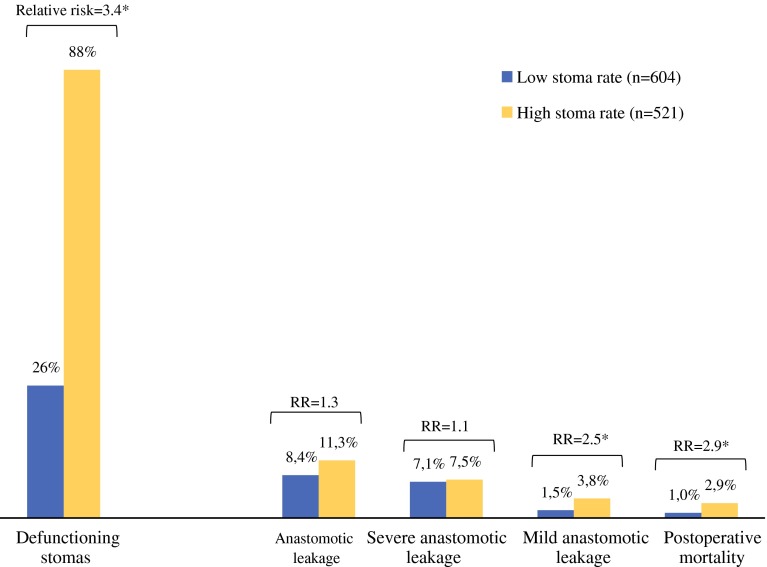
Comparison of outcomes between the groups identified as low and high stoma rates. Results with an *asterisk* are considered statistically significant (*p* < 0.05)

Eighteen hospitals with a total of 604 patients were identified as the group with low stoma rates. This group had a mean percentage of 26 % of patients with a defunctioning stoma. The group with high stoma rates consisted of 18 hospitals, which treated 521 patients in total, and had an 88 % mean defunctioning stoma rate (Fig. 3).

A slight difference in overall anastomotic leakage rates was found between groups, although this not statistically significant (8.4 vs.11.3 %; *p* = 0.11). Severe anastomotic leakage rates were similar in both groups (7.1 vs. 7.5 %; *p* = 0.95, while mild anastomotic leakage rates were significantly higher in the group with high stoma rates (1.5 vs. 3.8 %; *p* < 0.001). Postoperative mortality rates were significantly higher in the group with high stoma rates (2.9 vs. 1.0 %; *p* = 0.02). The remaining hospitals formed a group with intermediate stoma rates (67 %), and had outcomes between the low and high stoma groups (9.7 % anastomotic leakage, 1.7 % mortality).

Table 2 shows the results of univariate and multivariate analysis for factors contributing to the odds of being in the group with high stoma rates. The percentage of patients treated with short-course radiation therapy (SCRT) was higher in the group with high stoma rates, as was the percentage of patients treated in teaching hospitals.

**Table 2:** Univariate and multivariate analysis for factors contributing to being in the group with high stoma rates

Factor	Univariate		Multivariate	
	Cowboys [*n* (%)]	Chickens [*n* (%)]	OR^a^	95 % CI
Age, mean	66	66	0.99	0.98–1.01
Female sex	247 (41)	210 (40)	0.88	0.68–1.14
ASA score				
1	157 (30)	149 (30)	1.0	Ref
2	297 (56)	307 (60)	1.13	0.76–1.36
3+	79 (15)	52 (10)	0.81	0.55–1.30
Urgency				
Urgent operation	**18 (4)**	**4 (0.8)**	**0.29**	**0.09–0.89**
Preoperative surgery, yes	24 (4)	25 (5)	1.19	0.64–2.24
T stage (p)				
T0	22 (4)	32 (7)	1.0	Ref
T1	53 (9)	55 (11)	1.35	0.36–5.00
T2	193 (32)	165 (32)	1.02	0.29–3.61
T3	314 (52)	260 (50)	1.08	0.31–3.78
T4	22 (4)	9 (2)	0.62	0.14–2.74
Abdominal surgical history, yes	135 (22)	144 (28)	1.26	0.94–1.70
Tumor distance—anal verge, >10 cm	225 (37)	137 (33)	0.87	0.66–1.14
Neoadjuvant therapy, none	**171 (28)**	**100 (19)**	1.0	Ref
5 × 5				
5 × 5 Gy	**301 (50)**	**308 (60)**	**1.67**	**1.20**–**2.31**
Chemoradiation	132 (22)	133 (22)	1.13	0.72–1.69
Surgical treatment, laparoscopy	291 (50)	286 (55)	1.09	0.84–1.41
Hospital type, teaching	**259 (43)**	**269 (52)**	**2.88**	**2.04**–**4.10**
Volume				
<25	191 (32)	141 (27)	1.0	Ref
25–50	222 (36)	274 (53)	1.18	0.86–1.62
>50	**191 (32)**	**106 (20)**	**0.27**	**0.17**–**0.43**

Bolded data indicate statistically significant (*p* < 0.05)

*OR* odds ratio, *CI* confidence interval, *ASA* American Society of Anesthesiologists

^a^ORs display the odds for being in the group with high stoma rates

In addition, in multivariate analysis these patients had higher odds of being in the group with high stoma rates. In both univariate and multivariate analysis, urgent resections and volume were associated with a lower risk of being treated in a hospital with a high stoma rate (Table 2). Other case-mix factors, such as age, ASA score and tumor characteristics, were not statistically different in both groups.

## Discussion

### Overview of Findings

This study demonstrates a large variation between hospitals in treatment strategy concerning defunctioning stoma construction after surgical resection of rectal cancer, even after adjustment for relevant case-mix factors. Hospitals with a low threshold for defunctioning stoma construction after rectal cancer resection did not have lower anastomotic leakage rates in comparison with hospitals with an opposite strategy. Interestingly, mortality and anastomotic leakage rates requiring radiological drainage were even higher in hospitals with a high stoma rate. The latter may be partly due to the slight difference in SCRT between both groups. Although a direct correlation between clinically apparent anastomotic leakage and neoadjuvant therapy has not been demonstrated^4^,^23^–^26^ den Dulk et al. showed SCRT to be a limiting factor for reversal of a (secondary) constructed stoma, suggesting that it increases the risk for subclinical or mild anastomotic leakage.^10^

An explanation for the remarkable correlation between a risk adverse strategy and low hospital volume or teaching status cannot be provided within the scope of this article. These hospitals may possibly use other selection criteria for defunctioning stomas, or treat patients with an impaired condition which could not be adjusted in this study.

### Comparison with Other Studies

There is ongoing debate on the differences in treatment approach despite ample data describing the direct correlation between the rate of both defunctioning stomas on the one hand and anastomotic leakage and postoperative mortality on the other. The discussion focuses mainly on whether defunctioning stomas should be used routinely after low anterior resection to decrease anastomotic leakage rates. A meta-analysis from Hüser et al.^1^ mainly based on the results of a randomized controlled trial from Matthiessen et al.^2^ clarifies the advantage of a defunctioning stoma on lowering anastomotic leakage rates. This is confirmed by a number of retrospective studies.^4^,^27^–^29^ On the contrary, a study from Fielding et al.^30^ observed a higher leakage rate in patients with a defunctioning stoma (18 vs. 7 %) and suggested that surgeons with an individual anastomotic leakage rate less than 5 % do not need to create a defunctioning stoma at all. Both Enker et al. and Matthiessen et al. showed that a defunctioning stoma did not reduce the incidence of anastomotic leakage in patients undergoing low or ultralow anterior resection.^7^,^31^

### Strengths and Limitations of the Study

We retrospectively evaluated a prospectively maintained, population-based database to determine the association between the hospitals’ strategy regarding defunctioning stoma construction and postoperative outcome in rectal cancer. It could be argued that comparing patient outcomes for patients with and without a stoma is not valid because of confounding by indication; patients may have received a stoma because they were considered to be high risk patients and are therefore not comparable to patients who did not receive a defunctioning stoma. This bias could also explain the relatively high mortality in the group with high stoma rates; however, in our study this bias is largely overcome by comparing hospitals at both ends of the spectrum (either very high or very low defunctioning stoma rates). Defunctioning stoma rates of 88 and 26 %, respectively, reflect a strategic approach (standard with a stoma or standard without a stoma), which is only slightly based on individual decision making concerning patient characteristics. It is likely that only very high risk patients received a stoma in both groups, and very low-risk patients in both groups did not. For other patients, the decision was mainly based on the strategic approach of the hospitals. Therefore the method we used resembles a ‘pseudo randomization’. This is supported by the fact that baseline characteristics were similar for both groups in our study.

These findings are very useful for clinical practice because they strengthen the concept that the decision of stoma formation after anterior rectal resection cannot be standardized but requires careful evaluation of individual risk factors. Data represent current surgical practice at a population level since all hospitals participate in the DSCA and the percentage of eligible patients registered is over 90 %.

A limitation of this study is that analyses were performed at a hospital level, while the surgical strategy may differ between surgeons within a hospital. Information on a surgeons’ level is not available in the DSCA and individual volumes may be low, introducing more impact of chance variation in the analyses.

### Clinical Implications

Should we then be cowboys or chickens, if the latter does not necessarily result in better outcomes? The results confirm that the protective effect of a defunctioning stoma is probably most apparent in high-risk patients, while the additional benefit for the rest of the population is limited or even non-existent. There have been numerous studies identifying risk factors for anastomotic leakage.^9^–^13^ Dekker et al. developed and tested the colon leakage score (CLS) in which multiple risk factors were used to provide an objective prediction of the risk for anastomotic leakage.^32^ They found that only 20 % of their population could be considered as high risk. If we take into account the relative risk reduction of 64 % that was found in the randomized trial of Matthiessen et al. (reduction in anastomotic leakage from 28 to 10 %) for high-risk patients with an hypothetical a priori risk of anastomotic leakage of 20 %, this would mean an absolute risk reduction (ARR) of 12.8 %, and therefore eight defunctioning stomas would have to be constructed in order to prevent one anastomotic leak. In contrast, for patients with an a priori risk of 5 % (ARR 3.2 %), 31 defunctioning stomas would have to be created to prevent one leak.

It should therefore be kept in mind that stomas can induce morbidity, discomfort (quality of life), costs, and even mortality. Stomal complications cause readmission within 2 months after initial surgery in up to 17 % of all patients, mostly due to dehydratation.^9^,^11^,^33^,^34^ Even when a defunctioning stoma is constructed, there is still a considerable risk of (late) anastomotic leakage^2^,^4^,^35^–^37^ A recent study from our group on 1-year follow-up data shows a significant higher morbidity rate in patients with a defunctioning stoma when compared with patients without, due to unplanned readmissions (18 %) and reinterventions (12 %) caused by anastomotic leakage and drainage of abscesses.^37^ It is also recognized that 15–30 % of defunctioning stomas are never closed, resulting in a permanent stoma.^10^,^38^ Future studies are important to gain more evidence on the possible benefits of defunctioning stomas in high- and low-risk patients.

Finally, we advocate that patients’ preferences concerning the risk of morbidity and mortality of anastomotic leakage versus the consequences of a defunctioning stoma should be taken into account preoperatively.

## Conclusions

A high tendency towards defunctioning stoma construction in rectal cancer surgery did not result in lower overall anastomotic leakage or mortality rates. The optimal treatment strategy can probably be found in hospitals with both low stoma rates and favorable postoperative outcomes. It seems that hospitals with low stoma rates were better in selecting high-risk patients, and that stoma formation in more patients does not lead to better outcomes.

Adequate identification of high-risk patients should be the focus of future studies in order to facilitate decision making.
